# Bystander-witnessed cardiopulmonary resuscitation by nonfamily is associated with neurologically favorable survival after out-of-hospital cardiac arrest in Miyazaki City District

**DOI:** 10.1371/journal.pone.0276574

**Published:** 2022-10-21

**Authors:** Toshihiro Tsuruda, Takaaki Hamahata, George J. Endo, Yuki Tsuruda, Koichi Kaikita

**Affiliations:** 1 Faculty of Medicine, Department of Hemo-Vascular Advanced Medicine, Cardiorenal Research Laboratory, University of Miyazaki, Miyazaki, Japan; 2 Miyazaki City Fire Department, Miyazaki, Japan; 3 Faculty of Medicine, Endowed Department of Disaster/Emergency Medical Support, University of Miyazaki, Miyazaki, Japan; 4 Department of Emergency Medicine, Kobayashi City Hospital, Kobayashi, Japan; 5 Department of Clinical Pharmacy, Doshisha Women’s College of Liberal Arts, Kyotanabe, Japan; 6 Faculty of Medicine, Division of Cardiovascular Medicine and Nephrology, Department of Internal Medicine, University of Miyazaki, Japan; UT Health San Antonio: The University of Texas Health Science Center at San Antonio, UNITED STATES

## Abstract

**Background:**

Bystander intervention in cases of out-of-hospital cardiac arrest (OHCA) is a key factor in bridging the gap between the event and the arrival of emergency health services at the site. This study investigated the implementation rate of bystander cardiopulmonary resuscitation (CPR) and automated external defibrillator (AED) and 1-month survival after OHCA in Miyazaki prefecture and Miyazaki city district as well as compared them with those of eight prefectures in the Kyushu-Okinawa region in Japan. In addition, we analyzed prehospital factors associated with survival outcomes in Miyazaki city district.

**Methods:**

We used data from an annual report released by the Fire and Disaster Management Agency of Japan (n = 627,982) and the Utstein reporting database in Miyazaki city district (n = 1,686) from 2015 to 2019.

**Result:**

Despite having the highest rate of bystander CPR (20.8%), the 1-month survival rate (15.7%) of witnessed OHCA cases of cardiac causes in Miyazaki city district was comparable with that in the eight prefectures between 2015 and 2019. However, rates of survival (10.7%) in Miyazaki prefecture were lower than those in other prefectures. In 1,686 patients with OHCA (74 ± 18 years old, 59% male) from the Utstein reporting database identical to the 5-year study period in Miyazaki city district, binary logistic regression analysis demonstrated that age of the recipient [odds ratio (OR) 0.979, 95% confidential interval (CI) 0.964–0.993, *p* = 0.004)], witness of the arrest event (OR 7.501, 95% CI 3.229–17.428, *p* < 0.001), AED implementation (OR 14.852, 95% CI 4.226–52.201, *p* < 0.001), and return of spontaneous circulation (ROSC) before transport (OR 31.070, 95% CI 16.585–58.208, *p* < 0.001) predicted the 1-month survival with favorable neurological outcomes. In addition, chest compression at a public place (*p* < 0.001) and by nonfamily members (*p* < 0.001) were associated with favorable outcomes (*p* = 0.015).

**Conclusions:**

We found differences in 1-month survival rates after OHCA in the Kyushu-Okinawa region of Japan. Our results suggest that on-field ROSC with defibrillation performed by nonfamily bystanders who witnessed the event determines 1-month neurological outcomes after OHCA in Miyazaki city district. Continued education of citizens on CPR techniques and better access to AED devices may improve outcomes.

## Introduction

The link between the emergency medical dispatcher, the bystander who provides cardiopulmonary resuscitation (CPR), and the timely use of an automated external defibrillator (AED) is important for improving the survival of people who suffer out-of-hospital cardiac arrest (OHCA) [1]. Bystander CPR is the most modifiable factor for survival after OHCA [2,3]. However, fewer than 40% of adult patients with OHCA receive lay-person-initiated CPR, with AEDs used in fewer than 12% [4], whereas 47% of pediatric OHCA cases receive bystander CPR, with AED use attempted in 17% [5] before the emergency medical service (EMS) arrives. Meanwhile, the impact of transportation time from the scene to the arrival at the hospital on survival outcomes is debatable [6,7].

In 1991, Japan officially established the role of paramedics and enacted regulations, designating its workers as medical professionals and establishing that EMSs encompass both medical care on the field and transport to the medical facilities [8]. Paramedics are nationally licensed, and capabilities for resuscitation are secured for all prefectures. The Fire and Disaster Management Agency of Japan reported that the EMS-transported 126,271 cardiopulmonary arrest cases in 2019, of which 20% (n = 25,560) were witnessed by a bystander. Nationwide, the population-based registry system of OHCA provides evidence that neurological outcomes have improved over the years in Japan [9], but it also revealed a twofold regional variation in outcomes. Miyazaki prefecture had the 24th highest risk-adjusted survival rate and the 21st highest degree of favorable neurological outcomes 1 month after OHCA across 47 prefectures [10]. To spread knowledge regarding the CPR technique among citizens and to propose strategies for the health service department, we investigated the implementation rate of bystander CPR and AED, survival rate, and EMS transport time in Miyazaki city district and compared them with those in eight prefectures across the Kyushu-Okinawa region. In addition, we analyzed prehospital factors associated with 1-month survival with favorable neurological outcomes after OHCA in Miyazaki city district.

## Methods

### Ethical considerations

The study protocol conformed to the Declaration of Helsinki and was approved by the ethics committee of the University of Miyazaki (0–851). We used data from an anonymous database, so it was unnecessary to obtain written informed consent from each patient.

### Study design and data source

Kyushu island is the third largest island in Japan, and the Kyushu-Okinawa region comprises eight prefectures: Fukuoka (with a population of 5.1 million in 2021), Saga (812,000), Nagasaki (1.31 million), Kumamoto (1.74 million), Oita (1.12 million), Miyazaki (1.07 million), Kagoshima (1.59 million), and Okinawa (1.47 million). Miyazaki city is the most populated local city in Miyazaki prefecture. We enrolled all patients (0–103 years old) for whom emergency services were offered after OHCA in Miyazaki city district (Miyazaki city and the suburban areas of Kunitomi-cho and Aya-cho) between January 1, 2015, and December 31, 2019. The population of Miyazaki city district is 426,210 (September 2021), and Miyazaki City Fire Department provides basic life support ambulances staffed with ambulance technicians and paramedics in these areas. A total of ten EMSs are deployed in Miyazaki city district, and three crew members are usually staffed with one or two paramedics; EMS dispatches with four crew members in cases of OHCA.

We used data from an annual report released by the Fire and Disaster Management Agency of Japan from 2015 to 2019 [11], Japanese Association of Cardiovascular Intervention and Therapeutics (CVIT) in 2022 [12], and the Utstein reporting database [13] in Miyazaki city district from 2015 to 2019. Cardiac arrest was defined as the cessation of cardiac mechanical activity as confirmed by the absence of signs of circulation. An arrest was presumed to be of cardiac etiology unless it was known or likely to have been caused by trauma, submersion, drug overuse, asphyxia, exsanguination, or any other noncardiac cause as best determined by rescuers [13]. A bystander was defined as an individual who witnessed the collapse or who found the person unresponsive and activated the EMS system [14]. A witnessed cardiac arrest is one that is seen or heard by another person. Witnesses included family, nonfamily (acquaintance, colleague, or passerby), and EMS personnel. When EMS personnel performed CPR or attempted defibrillation, it was recorded as a resuscitation attempt by EMS personnel. The locations of the arrest included public places, offices, homes, and roads. Shockable rhythm refers to the first monitored rhythm, which was implemented by AED. The quality of the chest compressions was judged by a paramedic as effective or ineffective, subjectively but not qualitatively. EMS transport time is the interval between receiving an emergency call to the EMS dispatcher, EMS arrival on the field, and transport to the hospital. Return of spontaneous circulation (ROSC) was defined as a palpable pulse at common carotid artery and/or breathing, cough, or movement [13], which persists until hospital admission. We calculated the percentage of transport times that were less than 20 min, and EMS took 8.7 ± 4.0 (mean ± standard deviation) min from the dispatcher’s receipt to arrival on the field in this study. The cutoff point of the time interval for good Cerebral Performance Category (CPC) is 8 min between the EMS leaving the field and arrival at the hospital [6]. The physician responsible for the care of patients after successful resuscitation evaluated neurologic outcomes during a follow-up interview at 1 month using the following CPC scale: category 1, good cerebral performance; category 2, moderate cerebral disability; category 3, severe cerebral disability; category 4, coma or vegetative state; and category 5, death [13].

### Statistical analysis

All statistical analyses were performed using SPSS software, version 28 (IBM Corp., Armonk, NY, USA). Data were expressed as means ± standard deviation and number of patients. Continuous variables were compared by Student’s t-test, or one-way analysis of variance, followed by post-hoc Tukey’s honestly significant difference test. Categorical variables were compared using a χ^2^ test. Pearson correlation coefficient test was used to assess the relationship between the two parameters. Binary logistic regression analysis was performed to identify factors associated with 1-month survival with favorable neurological outcomes (CPC 1 or 2); odds ratios (ORs) and their 95% confidence intervals (CIs) were calculated. Potential confounding factors based on biological plausibility and previous studies were included in the multivariate analysis. These variables included age, sex, location of arrest, witnessed type, bystander CPR, defibrillation before EMS arrival, and ROSC prior to transport. All tests were two-sided, and a p-value less than 0.05 was statistically significant. Missing values were excluded.

## Results

### Bystander CPR and AED, 1-month survival, and EMS transport time in Miyazaki city district and the Kyushu-Okinawa region

**Fig 1:**
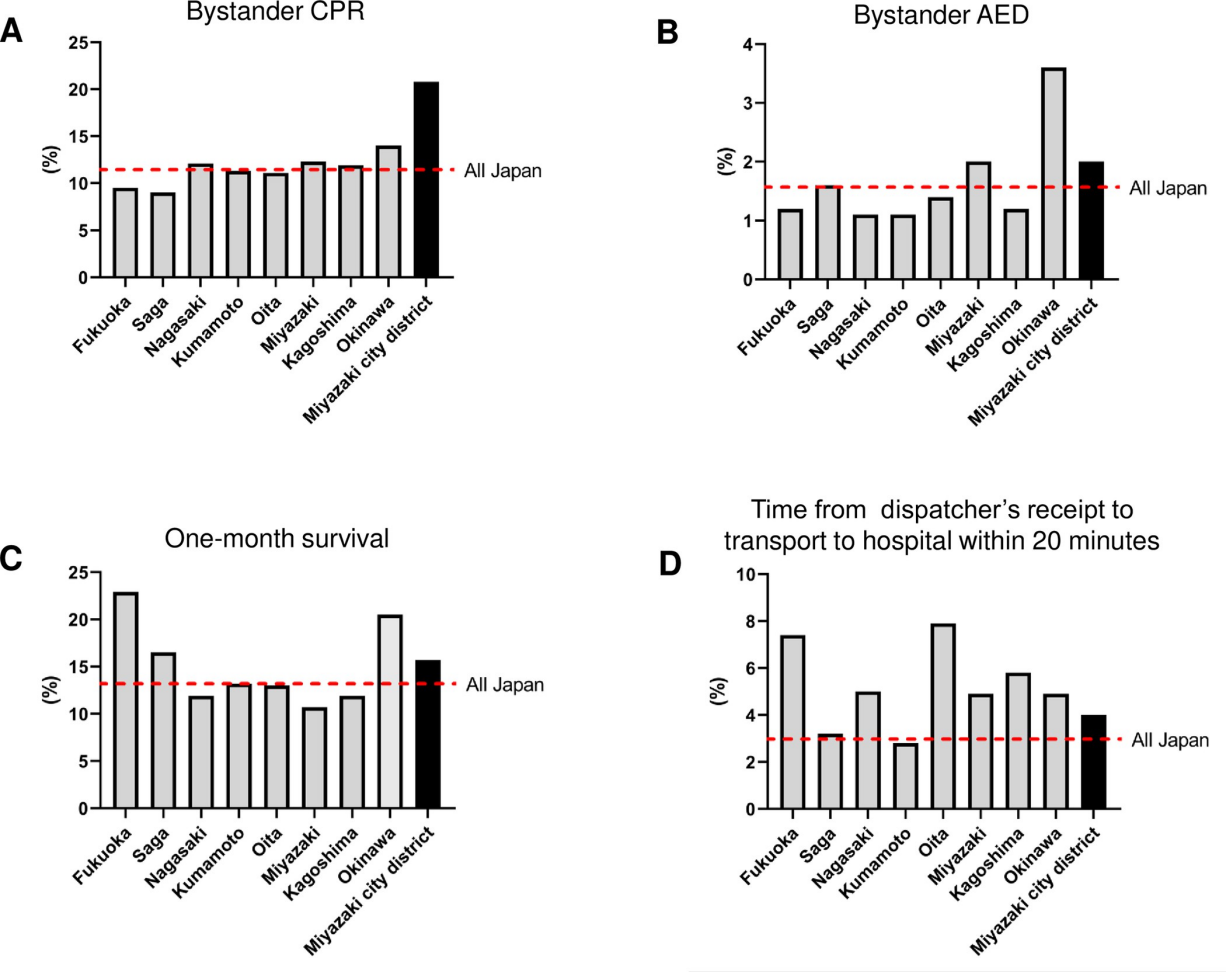
Percentage of bystander-witnessed CPR on OHCA due to cardiac causes **(A)**, and shocked AED **(B)** among EMS-transported patients with OHCA. **(C)** One-month survival rate for witnessed patients with OHCA of cardiac cause. **(D)** Percentage of EMS transport time that was less than 20 min. We accessed the annual report released by the Fire and Disaster Management Agency of Japan in 2020 and analyzed data from the Kyushu-Okinawa region and all over Japan between 2015 and 2019. The red dotted line indicates the percentage across 47 prefectures in Japan. Raw data are available in S1–S3 Tables.

Over the 5-year study period, 1,686 patients (999 males, 687 females) had emergency requests for OHCA at the EMS in Miyazaki city district. The mean age of the patients was 74 ± 18 (0–103) years, with 32 under 18 years old. Bystander-witnessed CPR for OHCA of cardiac causes was performed in 20.8% of the patients (350 of 1,686) (Fig 1A, S1 Table), and defibrillation was implemented in 2.0% (33 of 1,686) of the 1,686 EMS-transported patients with OHCA (Fig 1B, S1 Table) in Miyazaki city district. One-month survival regardless of neurological outcomes (CPC1-4) was 15.7% (71 of 452) in bystander-witnessed OHCA of cardiac causes (home recuperation, 22; outpatient, 17; transfer to rehabilitation hospital, 8; and remained admitted to the hospital, 24) (Fig 1C, S2 Table); meanwhile, the percentage of EMS transport trips of <20 min was 4.03% (2,982 of 73,931) in Miyazaki city district (Fig 1D, S3-1 Table in S3 Table). Each value was comparable to those from the average throughout Japan and other prefectures in the Kyushu-Okinawa region. Bystander-witnessed CPR ranged from 9.0% (379 of 4,231) in Saga prefecture to 14.0% (876 of 6,258) in Okinawa prefecture; bystander defibrillation ranged from 1.1% (75 of 6,612) in Nagasaki prefecture and 1.1% (92 of 8,548) in Kumamoto prefecture to 3.6% (227 of 6,258) in Okinawa prefecture. One-month survival was highest in Fukuoka prefecture [22.9% (683 of 2,979)], and an EMS transport time of <20 min was greatest in Oita prefecture [7.9% (19,816 of 249,917)] within the Kyushu-Okinawa region (S3-2 Table in S3 Table). Rates of survival in Miyazaki prefecture were 10.7% (117 of 1,091) of those in other prefectures despite comparable rates of bystander CPR (12.3%, 676 of 5,501) and defibrillation (2.0%, 111 of 5,501) (Fig 1A–1C, S1 and S2 Tables).

In an annual report released in 2020, the Fire and Disaster Management Agency of Japan documented the number of hospitals announced as emergency hospitals per 100,000 population across the eight prefectures (3.0 in Fukuoka, 5.5 in Saga, 4.1 in Nagasaki, 4.9 in Kumamoto, 4.9 in Oita, 5.6 in Miyazaki, 6.1 in Kagoshima, and 1.7 in Okinawa). The numbers were negatively associated with 1-month survival outcomes (*r* = −0.771, *p* = 0.025; S1A Fig). In contrast, the availability of facilities certified by the Japanese Association of Cardiovascular Intervention and Therapeutics (CVIT) [12] per 100,000 population in 2022 (0.73 in Fukuoka [37 of 5.1 million], 0.49 in Saga [4 of 0.812 million], 0.69 in Nagasaki [9 of 1.31 million], 0.46 in Kumamoto [8 of 1.74 million], 0.45 in Oita [5 of 1.12 million], 0.28 in Miyazaki [3 of 1.07 million], 0.63 in Kagoshima [10 of 1.59 million], and 0.68 in Okinawa [10 of 1.47 million]) tended to be associated with 1-month survival outcomes (*r* = 0.592, *p* = 0.122; S1B Fig).

### Characteristics of patients with OHCA in the Miyazaki city district

Table 1 shows the characteristics of patients who received bystander CPR and those who did not receive resuscitation by a bystander.

**Table 1:** Background of those who received resuscitation, and those who did not receive resuscitation by a bystander.

	Resuscitation attempts	Resuscitation Not attempted	*p*-value
**Number**	868	818	
**Age (range)**	74 ± 19 (0–101)	73 ± 17 (0–103)	0.405^†^
**Sex**			0.021^‡^
Male	491	508	
Female	377	310	
**Location of arrest**			< 0.001^‡^
Public place	317	139	
Office	22	11	
Home	482	584	
Road	33	46	
Other	14	38	
**Cause of arrest**			0.037^‡^
Noncardiac	518	447	
Cardiac	350	371	
**Arrest witnessed**	520	409	< 0.001^‡^
**Witness, type**			< 0.001^‡^
Family	164	176	
Nonfamily	46	35	
EMS personnel	4	133	
Others	142	69	
**AED attempts**	105	0	< 0.001^‡^
**AED shocks**	33	0	< 0.001^‡^
**Outcomes**			
CPC (1: 2: 3: 4: 5)	36: 1: 5: 8: 818	30: 4: 11: 13: 760	0.169^‡^

Data are listed either as means ± standard deviations or as number of patients.

^†^Unpaired *t*-test; ^‡^chi-squared test. AED, automated external defibrillator; CPC, Cerebral Performance Category; EMS, emergency medical service. “Outcomes” refers to neurologic outcomes documented during a follow-up interview at 1 month according to the following CPC scale: Category 1, good cerebral performance; category 2, moderate cerebral disability; category 3, severe cerebral disability; category 4, coma or vegetative state; and category 5, death.

Bystander CPR was performed predominantly on females (χ^2^ = 5.345, *p* = 0.021), at a public place or office (χ^2^ = 94.726, *p* < 0.001), having an arrest that was not witnessed (χ^2^ = 16.710, *p* < 0.001), having an arrest event that was of cardiac cause (χ^2^ = 4.357, *p* = 0.037), and by nonfamily (an acquaintance or colleague) (χ^2^ = 168.509, *p* < 0.001). In addition, 12% (105 of 868) had attempted AED (χ^2^ = 105.523, *p* < 0.001), and 4% (33 of 868) were shocked with AED (χ^2^ = 31.720, *p* < 0.001). Attempted bystander CPR did not affect the 1-month survival with favorable neurological outcomes in 1,686 patients with OHCA (χ^2^ = 6.441, *p* = 0.169).

### Characteristics of patients with OHCA who achieved ROSC

Table 2 shows the characteristics patients who achieved ROSC categorized into prior to transport, during transport, and arrival at hospital. ROSC prior to transport was associated with younger age (*p* < 0.001), shorter time to achieve ROSC since dispatcher’s receipt (*p* < 0.001), arrest witnessed by nonfamily bystanders (χ^2^ = 152.344, *p* < 0.001), attempted AED (χ^2^ = 42.411, *p* < 0.001), shocked AED (χ^2^ = 91.901, *p* < 0.001), more public place, fewer events at home (χ^2^ = 37.130, *p* < 0.001), and 1-month favorable neurological outcomes (χ^2^ = 592.545, *p* < 0.001).

**Table 2:** Background of those who returned circulation spontaneously after out-of-hospital cardiac arrest.

	ROSC			No ROSC	*p*-value
	**Before transport**	**During transport**	**Arrival at hospital**		
**Number**	90	111	8	1477	
**Age (range)**	66 ± 21 (1–94)^*a*, *b*, *c*^	75 ± 16 (12–101)	83 ± 14 (58–101)	74 ±18 (0–103)	< 0.001^†^
**Sex (M: F)**	63: 27	62: 49	6: 2	868: 609	0.122^‡^
**Dispatcher’s receipt to ROSC, min (range)**	18 ± 14 (0–79)^*d*, *e*^	35 ± 18 (6–112)	31 ± 5 (23–41)		< 0.001^†^
**Location of arrest** Public place Office Home Road Other	39 5 36 8 2	28 4 73 2 4	5 0 3 0 0	384 24 954 69 46	< 0.001^‡^
**Cause of arrest** Noncardiac Cardiac	36 54	51 60	0 8	634 843	0.081^‡^
**Arrest, witnessed**	70	74	7	606	< 0.001^‡^
**Witness, type** Family Nonfamily EMS personnel Others None	16 13 12 30 19	37 7 17 13 37	2 0 2 3 1	285 61 106 168 857	< 0.001^‡^
**Resuscitation attempt**	56	58	6	748	0.095^‡^
**AED attempts**	19	12	0	74	< 0.001^‡^
**AED shocks**	14	2	0	17	< 0.001^‡^
**Outcomes** CPC (1: 2: 3: 4: 5)	42: 1: 8: 4: 35	7: 2: 1: 7: 94	0: 0: 0: 0: 8	17: 2: 7: 10: 1 441	< 0.001^‡^

Data are listed either as means ± standard deviations or as number of patients. ^†^One-way analysis of variance, followed by post-hoc Tukey’s honestly significant difference test (*a* [before transport vs. during transport], *p* = 0.002; *b* [before transport vs. at the hospital], *p* = 0.043; *c* [before transport vs. at No ROSC], *p* < 0.001; *d* [before transport vs. during transport, *p* < 0.001; and *e* [before transport vs. at the hospital], p = 0.073); ^‡^chi-squared test. ROSC, return of spontaneous circulation; AED, automated external defibrillator; CPC, Cerebral Performance Category. “Outcomes” refers to neurologic outcomes documented during a follow-up interview at 1 month according to the following CPC scale: Category 1, good cerebral performance; category 2, moderate cerebral disability; category 3, severe cerebral disability; category 4, coma or vegetative state; and category 5, death.

### Predictors of 1-month survival with favorable neurological outcomes

Binary logistic regression analysis showed that recipient age [OR 0.979, 95% CI 0.964–0.993, *p* = 0.004)], witness of an arrest event (OR 7.501, 95% CI 3.229–17.428, *p* < 0.001)], defibrillation before EMS arrival on the field (OR 14.852, 95%CI 4.226–52.201, *p* < 0.001), and ROSC before transport (OR 31.070, 95%CI 16.585–58.208, *p* < 0.001) were independent predictors determining 1-month survival with favorable neurological outcomes (CPC 1 or CPC 2) after OHCA. Sex, location of arrest, or bystander CPR did not influence the outcome (Table 3).

**Table 3:** Binary logistic regression analysis to determine the favorable neurological outcomes (CPC 1 or 2) in 1,686 patients with OHCA.

Covariates	Odd Ratio (95% Confidence Interval)	*p*-value
Age	0.979 (0.964–0.993)	0.004
Arrest witness	7.501 (3.229–17.428)	< 0.001
Defibrillation before EMS arrival	14.852 (4.226–52.201)	< 0.001
ROSC before transport	31.070 (16.585–58.208)	< 0.001

Binary logistic regression analysis was constructed with forced entry in 1,686 patients with out-of-hospital cardiac arrest (OHCA). Dependent variables were scored 1 (Cerebral Performance Category [CPC] 1 or 2) or 0 (CPC 3 + CPC 4 + CPC 5). Covariables included in the analysis were age, sex (scored 1 for male and 2 for female), location of arrest (1, home; 0, other places), witnessed arrest (1, yes; 0, no); bystander cardiopulmonary resuscitation (CPR) (1, yes; 0, no); defibrillation before arrival of emergency medical service (EMS; 1, yes; 0, no); and return of spontaneous circulation (ROSC) before transport (1, yes; 0, no).

### Background of patients with OHCA who were given defibrillation with public-access AED

Defibrillation with AED was more often implemented on younger patients with OHCA (*p* = 0.033), on males (χ^2^ = 6.894, *p* = 0.009), at public places (χ^2^ = 44.730, *p* < 0.001), and in those who had OHCA due to cardiac causes (χ^2^ = 9.032, *p* = 0.003). The neurological outcomes were better in patients who received AED than in those who were not shocked (χ^2^ = 151.715, *p* < 0.001) (Table 4).

**Table 4:** Background of patients with OHCA who were given defibrillation with public-access AED.

	Use of AED	No Use of AED	*p*-value
**Number**	33	835	
**Age**	67 ± 19	74 ± 19	0.033^†^
**Sex**			0.007^‡^
Male	26	465	
Female	7	370	
**Location of arrest**			< 0.001^‡^
Public place	23	296	
Office	5	17	
Home	1	477	
Road	3	29	
Other	1	16	
**Cause of Arrest**			0.005^‡^
Noncardiac	5	345	
Cardiac	28	490	
**Outcomes**			
CPC (1: 2: 3: 4: 5)	15: 0: 1: 0: 17	21: 1: 4: 8: 801	< 0.001^‡^

Data are listed either as means ± standard deviations or as number of patients.

^†^Unpaired *t*-test; ^‡^chi-squared test. CPC, Cerebral Performance Category; AED, automated external defibrillator. “Outcomes” refers to neurologic outcomes documented during a follow-up interview at 1 month according to the following CPC scale: Category 1, good cerebral performance; category 2, moderate cerebral disability; category 3, severe cerebral disability; category 4, coma or vegetative state; and category 5, death.

### Quality of chest compression on 1-month survival with favorable neurological outcomes

The quality of chest compression was judged as effective in 48% (414 of 868) of patients with OHCA. Effective chest compression was implemented on younger patients (*p* = 0.017), at public places (χ^2^ = 53.180, *p* < 0.001), and by nonfamily (χ^2^ = 37.946, *p* < 0.001), leading to greater 1-month favorable neurological outcomes (χ^2^ = 12.310, *p* = 0.015) (Table 5).

**Table 5:** Quality of chest compression at 1-month neurological outcomes.

	CC Effective	CC Ineffective	*p*-value
**Number**	414	282	
**Age**	73 ± 19	77 ± 16	0.017^†^
**Sex**			0.119^‡^
Male	242	148	
Female	172	134	
**Location of arrest**			< 0.001^‡^
Public place	180	59	
Office	11	5	
Home	198	212	
Road	18	3	
Other	7	3	
**Witness**			< 0.001^‡^
Family	69	67	
Nonfamily	36	2	
EMS personnel	2	3	
Unknown	82	27	
**Outcomes**			
CPC (1: 2: 3: 4: 5)	19: 1: 5: 5: 384	2: 1: 0: 4: 275	0.015^‡^

Data are listed either as means ± standard deviations or as number of patients.

^†^Unpaired *t*-test; ^‡^chi-squared test. CC, chest compressions; CPC, Cerebral Performance Category. “Outcomes” refers to neurologic outcomes documented during a follow-up interview at 1 month according to the following CPC scale: Category 1, good cerebral performance; category 2, moderate cerebral disability; category 3, severe cerebral disability; category 4, coma or vegetative state; and category 5, death.

## Discussion

We report the regional disparity in the implementation rate of bystander CPR and AED as well as EMS transport time and 1-month survival outcomes in the Kyushu-Okinawa region. In addition, we describe that high-quality bystander CPR and defibrillation witnessed by nonfamily bystanders, and the on-field ROSC are associated with 1-month survival with favorable neurological outcomes after OHCA in Miyazaki city district. Based on these analyses, we propose strategies to improve the survival rate with favorable neurological outcomes after OHCA.

### Emergency medical institutions and survival outcomes

The primary hypothesis was that increased rate of bystander CPR improves rates of 1-month survival with favorable neurological outcomes. However, in Fukuoka prefecture, which had lower rates of bystander CPR, survival outcomes were better, and in Miyazaki prefecture, which had higher rates of bystander CPR, survival outcomes were worse. Our data indicated that EMS transport time does not always affect 1-month survival, as previously reported [7]. Interestingly, the number of medical facilities declared as emergency hospitals was negatively associated with improved 1-month survival outcomes (S1A Fig). Our study showed that 71 patients with OHCA of cardiac causes survived until 1 month; however, it was not the case in noncardiac causes. As shown in S1B Fig, survival outcomes of OHCA might partly depend on the presence of medical facilities capable of postcardiac arrest care (e.g., emergency coronary angiography for ST elevation myocardial infarction and targeted temperature management). There were lower 1-month survival outcomes in Miyazaki prefecture than Miyazaki city district, a central city and 2 of 3 certified facilities by Japanese Association of Cardiovascular Intervention and Therapeutics (CVIT) are in Miyazaki city district.

### Barriers to performing bystander CPR

Bystander intervention in cases of OHCA is a key factor that bridges the gap between the event and EMS arrival on the field [2,3]. It is important to analyze the factors associated with survival after bystander CPR. In this study, witnessing the arrest incident is an independent predictor associated with favorable outcomes after OHCA at 1 month. However, 50% (409 of 818) of patients with OHCA were not given CPR despite bystanders witnessing the arrest event. We speculate that these 409 OHCA include cases potentially amenable to AED. Bystanders have a complex behavioral response and barriers against initiating and continuing CPR. First, fear of causing harm, visible signs of vomit/blood, and lack of CPR skills might make a person hesitate to initiate bystander CPR [15,16]. Second, passersby of low income, low education, and race differences are typically unwilling to perform CPR [5,17]. Third, females were less likely to receive public-access AED, and bystanders are unwilling to undress females because of worries about misunderstandings, social norms, and sexual assault [18]. Thus, people who ultimately receive CPR and defibrillation after a witnessed cardiac arrest seemingly depends on where the arrest happens [19].

### Quality of chest compression until the arrival of EMS

Although Miyazaki city district had the highest rate of bystander CPR, 1-month survival rates were comparable with those in other prefectures within the Kyushu-Okinawa regions. In accordance with previous studies [20,21], we demonstrated that the quality of chest compression until EMS arrival on the field was associated with 1-month survival and favorable neurological outcomes. The likelihood of receiving CPR at home decreases with age and is lower compared with that in public [22]. Our data suggest that chest compression performed by family members at home was ineffective [16]. Although we could not determine who performed CPR at home, spouses may most likely witness the event, and their lack of confidence (e.g., early warning signs and symptoms) may delay CPR initiation [23]. We speculate that the characteristics of patients with OHCA, such as their age and predisposing diseases, may affect the decision of family members to perform CPR [24]. Maintaining the quality of CPR is another concern of older bystanders [25]. Guidelines recommend chest compressions 5–6 cm in depth at a rate of 100–120 per minute [26]; however, longer time periods decrease performance [27] such that the first 2 min of chest compressions are effective, but subsequent fatigue reduces their quality [28]. The provision of dispatcher-assisted CPR (known as telephone CPR) [1] and a smartphone application with an animation to explain the CPR technique could help maintain the quality of CPR [29,30]. EMSs in Miyazaki city district have two types of mechanical chest compression devices [LUCAS® (Stryker Medical, Portage, MI, US) and Clover3000 (Koken Co., Ltd, Tokyo, Japan)], which paramedics use during transport to the hospital. Sheraton et al. [31] reported that the use of mechanical chest compression was not associated with improved rates of ROSC during OHCA regardless of the presenting rhythm in meta-analysis and trial sequential analysis. Our data support the report by Wampler et al. [32] who showed that early successful ROSC via prehospital care is critical for improved survival.

### Access to public AED

Each minute that defibrillation is delayed decreases survival in shockable patients with OHCA [33–35]. Our study demonstrated that 63% (1,066 of 1,686) of arrests occurred at home. Inaccessibility of AED from one’s house delays the timing of defibrillation [36]. In this analysis, one patient had successful implementation of AED at home as the device was placed as a public-access AED inside the condominium building. People may not know where AEDs are placed near their living areas. AED locations appear when you ask your smartphone, “where is the nearest AED?” However, terms, such as “electrical shock” (e.g., denki-shokku or jo-sai-do-ki in Japanese), instead of AED were not able to detect the location of AEDs on the map. Another concern is that AEDs are typically placed inside the door of most public facilities, and they are not available for use outside of office hours.

### Proposals to further improve survival after OHCA

It is important to train first witnesses in dealing with sudden cardiac arrests throughout the community. First, this study suggests that continuing to educate citizens on CPR techniques and basic support (i.e., hands-only CPR, brief video kits, mobile applications, or social media broadcasting) [30] by community health services are necessary to improve survival with favorable neurological outcomes after OHCA. CPR skills decay within weeks to months after training [37]. We continue to educate middle school- and high school-aged children and school staff regularly to prevent sudden death of students with hypertrophic cardiomyopathy and coronary artery abnormalities [38]. There might also be benefits in educating family members of those at highest risk of coronary artery disease, children with congenital heart disease [19], and those at risk of asphyxia [38]. More importantly, widespread education of citizens to retain their CPR skills is needed, and this might include people who are not interested in resuscitation. Citizens take CPR training when they obtain a driver’s license in Japan. We propose that CPR training programs should be repeated at every license renewal. Sex-related barriers are another serious concern. We propose the use of female training manikins in CPR training courses. AED instruments that can be used with clothes on should also be developed. Second, public-access AEDs should be strategically located to easily deliver them within 5 min of the arrest event [39,40], and emergency dispatch guidance to inform the location of the nearest available defibrillator is expected to increase the use of AED for bystanders [14]. For instance, there are more than 140 convenience stores in Miyazaki city district. Lent fee is estimated to be approximately 5,000 yen per unit per month, and we need to discuss drawing up a budget to place AEDs with the city government.

### Limitations

There are several limitations in this analysis. EMS personnel are required to complete assessments and assign documentation using an Utstein-style template. Additionally, there was some missing data in the database. First, the monitored rhythm detected by AEDs was not documented. Second, the quality of chest compressions was not assessed objectively. In addition, it was not evaluated in 20% (172 of 868) of patients with OHCA given that rescuers suspended resuscitation when EMS arrived or failed to record this information. Third, the number and sex of bystanders and family members were not available in the datasheet. CPR performance changes according to the number of bystanders [3], and a simulator study demonstrated that women had a slower tempo of over 3 min of chest compressions than men [41].

## Conclusion

There was regional disparity in 1-month survival after OHCA in the Kyushu-Okinawa region, Japan. Patients living at Miyazaki prefecture had lower survival outcomes despite higher bystander CPR rate than those living at Fukuoka prefecture, suggesting the lack of well-trained postcardiac arrest care medical facilities. In addition, our results demonstrated that favorable 1-month survival was associated with younger age, witness of the arrest event, implementation of AED, and ROSC before transport. Public location of CPR and implementation by nonfamily members were also associated with better outcomes. These data suggest that continuing CPR education, use of female dummies in training scenarios, and better access to AED devices can increase early successful ROSC and improve survival outcomes after OHCA in Miyazaki city district.

## Supporting information

S1 Fig Relationship between the numbers of medical facilities announced as emergency hospitals released from the Fire and Disaster Management Agency of Japan (A) and facilities certificated by the Japanese Association of Cardiovascular Intervention and Therapeutics (CVIT) (B) and 1-month survival outcomes.
(TIF)

S1 Table Bystander CPR in patients with OHCA of cardiac cause, and bystander AED in all EMS- transported patients with OHCA in Miyazaki city district, and Kyushu-Okinawa regionbetween 2015 and 2019. (PPTX)

S2 Table One-month survival rate in OHCA patients of cardiac cause in Miyazaki city district and Kyushu-Okinawa region between 2015 and 2019. (PPTX)

S3 Table EMS transport time in Miyazaki city district (S3-1) and all over Japan (S3-2). (XLSX)
