# Supplementary figures and images for: Bystander-witnessed cardiopulmonary resuscitation by nonfamily is associated with neurologically favorable survival after out-of-hospital cardiac arrest in Miyazaki City District
Source: PLoS One. 2022 Oct 21;17(10):e0276574. doi: 10.1371/journal.pone.0276574 (PMC9586377; doi:10.1371/journal.pone.0276574)

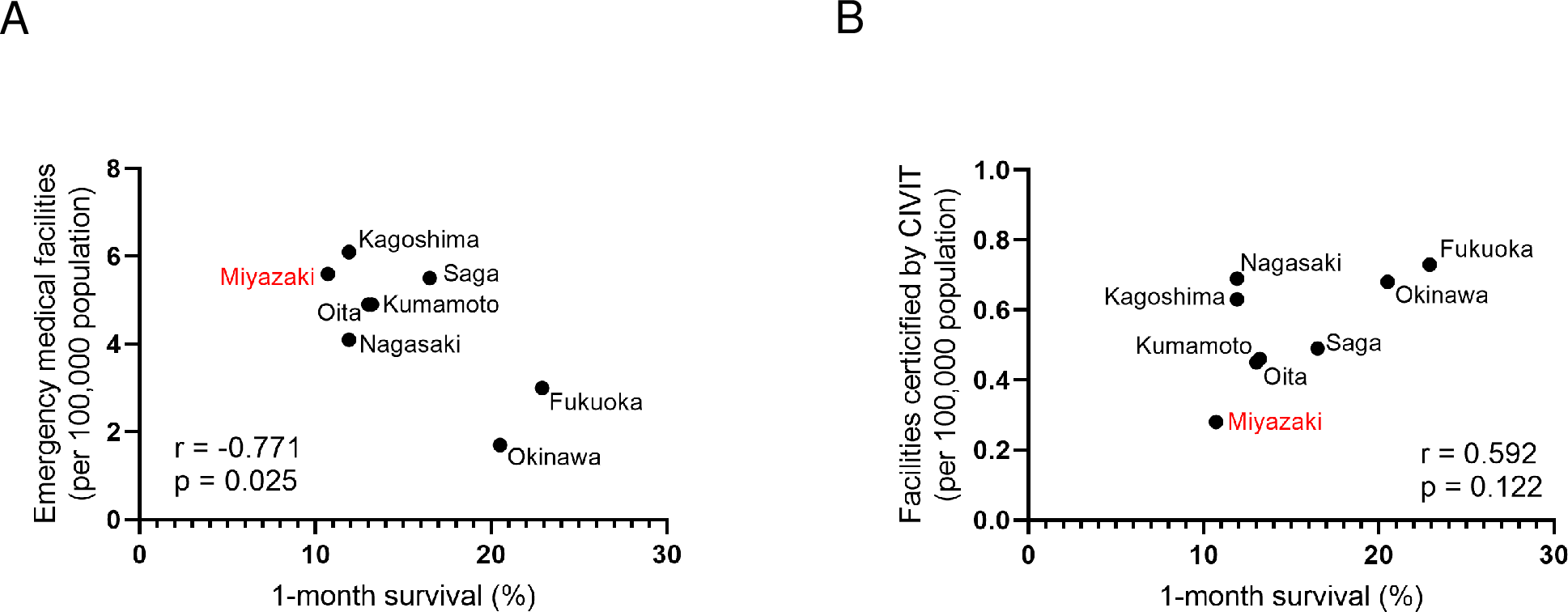

Supplement: S1 Fig — Relationship between the numbers of medical facilities announced as emergency hospitals released from the Fire and Disaster Management Agency of Japan (A) and facilities certificated by the Japanese Association of Cardiovascular Intervention and Therapeutics (CVIT) (B) and 1-month survival outcomes. (TIF) [file pone.0276574.s001.tif]
