# Supplementary material for: Bystander-witnessed cardiopulmonary resuscitation by nonfamily is associated with neurologically favorable survival after out-of-hospital cardiac arrest in Miyazaki City District
Source: PLoS One. 2022 Oct 21;17(10):e0276574. doi: 10.1371/journal.pone.0276574 (PMC9586377; doi:10.1371/journal.pone.0276574)
Supplement: S1 Table — (PPTX) [file pone.0276574.s002.pptx]

## Slide 1
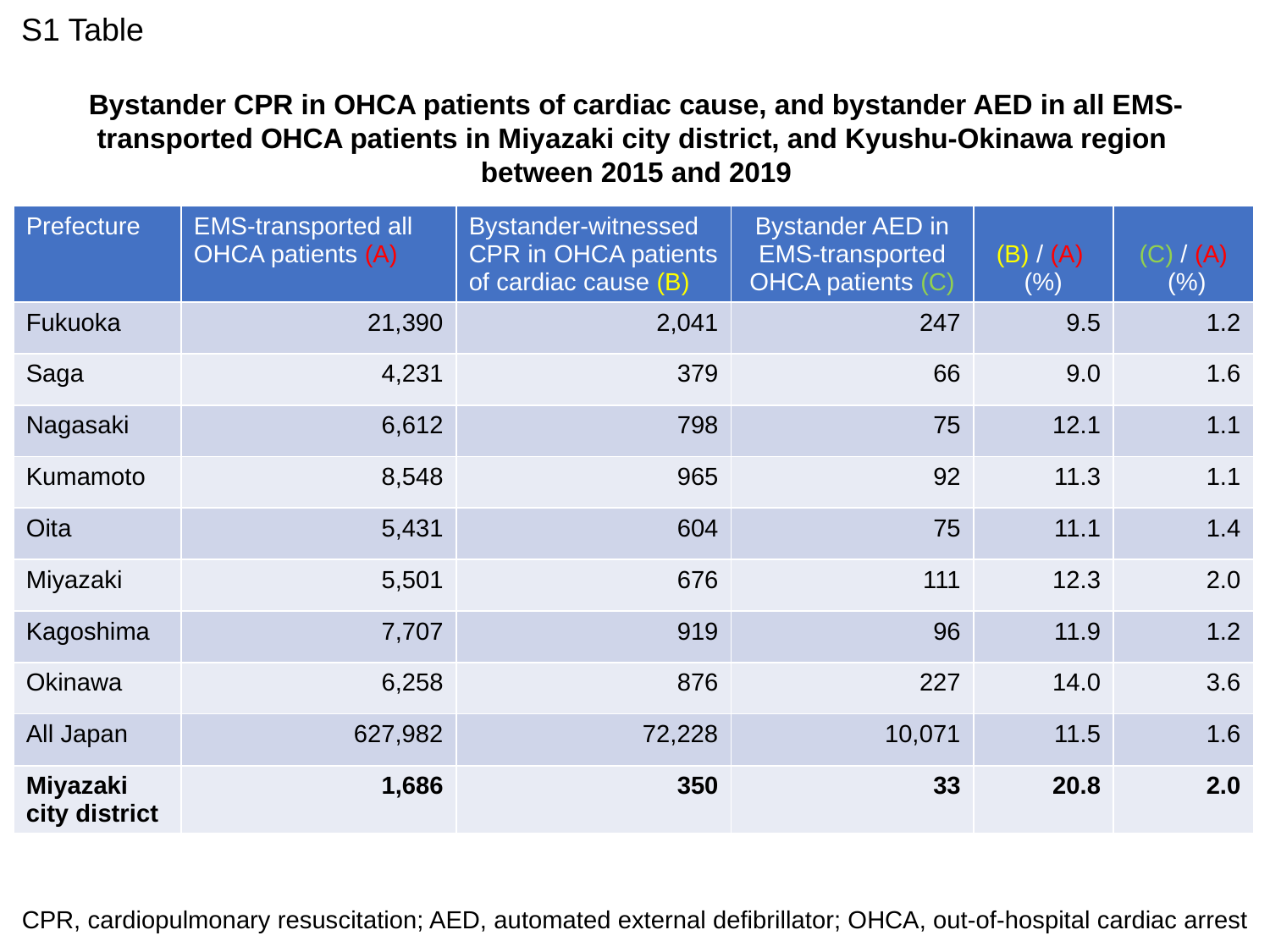

S1 Table
Bystander CPR in OHCA patients of cardiac cause, and bystander AED in all EMS- transported OHCA patients in Miyazaki city district, and Kyushu-Okinawa region
between 2015 and 2019
| Prefecture | EMS-transported all OHCA patients (A) | Bystander-witnessed CPR in OHCA patients of cardiac cause (B) | Bystander AED in EMS-transported OHCA patients (C) | (B) / (A) (%) | (C) / (A) (%) |
| --- | --- | --- | --- | --- | --- |
| Fukuoka | 21,390 | 2,041 | 247 | 9.5 | 1.2 |
| Saga | 4,231 | 379 | 66 | 9.0 | 1.6 |
| Nagasaki | 6,612 | 798 | 75 | 12.1 | 1.1 |
| Kumamoto | 8,548 | 965 | 92 | 11.3 | 1.1 |
| Oita | 5,431 | 604 | 75 | 11.1 | 1.4 |
| Miyazaki | 5,501 | 676 | 111 | 12.3 | 2.0 |
| Kagoshima | 7,707 | 919 | 96 | 11.9 | 1.2 |
| Okinawa | 6,258 | 876 | 227 | 14.0 | 3.6 |
| All Japan | 627,982 | 72,228 | 10,071 | 11.5 | 1.6 |
| Miyazaki city district | 1,686 | 350 | 33 | 20.8 | 2.0 |
CPR, cardiopulmonary resuscitation; AED, automated external defibrillator; OHCA, out-of-hospital cardiac arrest
