# Supplementary material for: Bystander-witnessed cardiopulmonary resuscitation by nonfamily is associated with neurologically favorable survival after out-of-hospital cardiac arrest in Miyazaki City District
Source: PLoS One. 2022 Oct 21;17(10):e0276574. doi: 10.1371/journal.pone.0276574 (PMC9586377; doi:10.1371/journal.pone.0276574)
Supplement: S2 Table — (PPTX) [file pone.0276574.s003.pptx]

## Slide 1
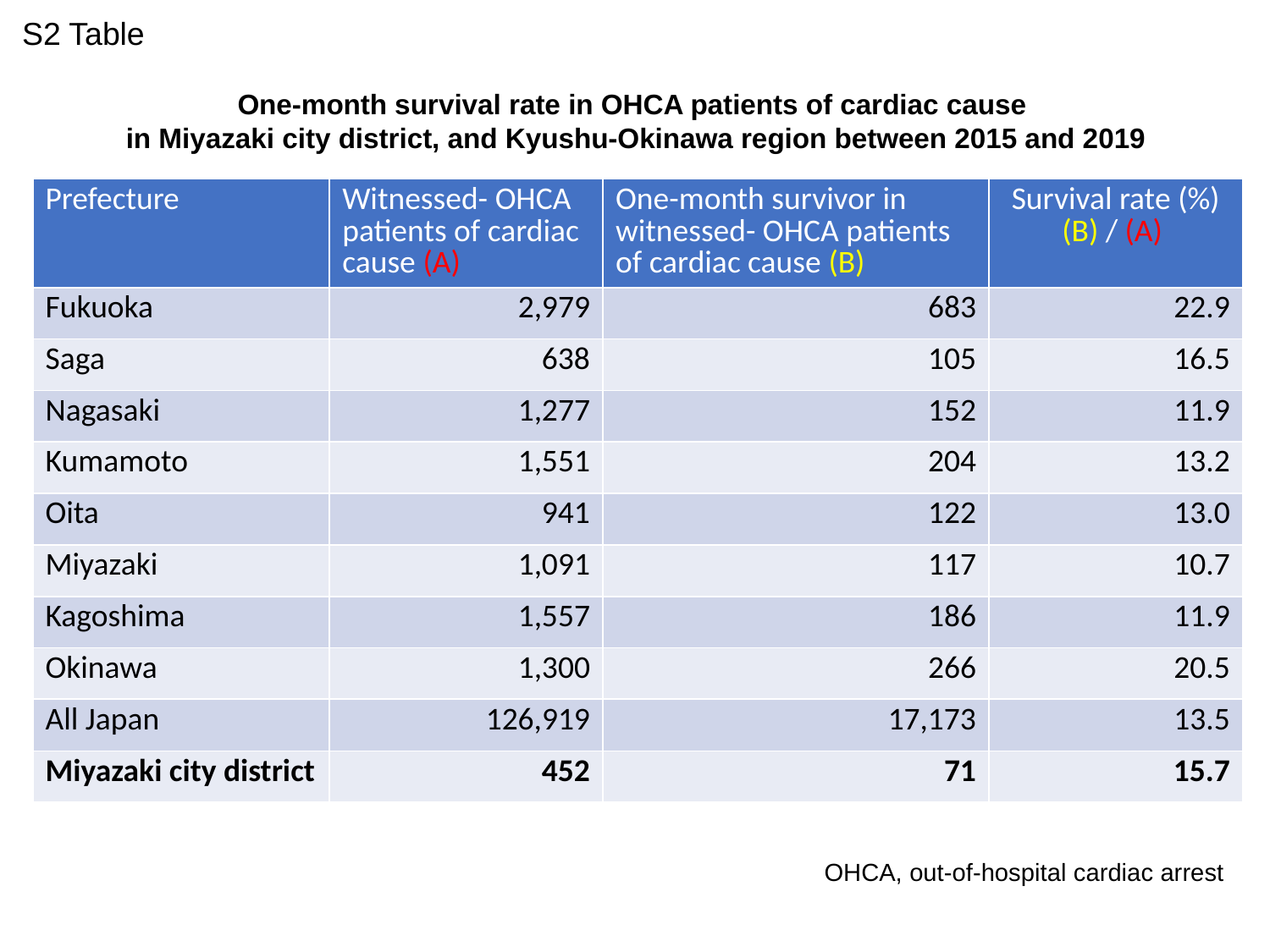

S2 Table
One-month survival rate in OHCA patients of cardiac cause
in Miyazaki city district, and Kyushu-Okinawa region between 2015 and 2019
| Prefecture | Witnessed- OHCA patients of cardiac cause (A) | One-month survivor in witnessed- OHCA patients of cardiac cause (B) | Survival rate (%) (B) / (A) |
| --- | --- | --- | --- |
| Fukuoka | 2,979 | 683 | 22.9 |
| Saga | 638 | 105 | 16.5 |
| Nagasaki | 1,277 | 152 | 11.9 |
| Kumamoto | 1,551 | 204 | 13.2 |
| Oita | 941 | 122 | 13.0 |
| Miyazaki | 1,091 | 117 | 10.7 |
| Kagoshima | 1,557 | 186 | 11.9 |
| Okinawa | 1,300 | 266 | 20.5 |
| All Japan | 126,919 | 17,173 | 13.5 |
| Miyazaki city district | 452 | 71 | 15.7 |
OHCA, out-of-hospital cardiac arrest
